# Osmolyte cooperation affects turgor dynamics in plants

**DOI:** 10.1038/srep30139

**Published:** 2016-07-22

**Authors:** Alfredo Argiolas, Gian Luigi Puleo, Edoardo Sinibaldi, Barbara Mazzolai

**Affiliations:** 1 Center for Micro-BioRobotics, Istituto Italiano di Tecnologia, Viale Rinaldo Piaggio 34, 56025 Pontedera, Italy; 2 The BioRobotics Institute, Scuola Superiore Sant’Anna, Viale Rinaldo Piaggio 34, 56025 Pontedera, Italy

## Abstract

Scientists have identified turgor-based actuation as a fundamental mechanism in plant movements. Plant cell turgor is generated by water influx due to the osmolyte concentration gradient through the cell wall and the plasma membrane behaving as an osmotic barrier. Previous studies have focused on turgor modulation with respect to potassium chloride (KCl) concentration changes, although KCl is not efficiently retained in the cell, and many other compounds, including L-glutamine (L-Gln) and D-glucose (D-Glc), are present in the cytosol. In fact, the contributions of other osmolytes to turgor dynamics remain to be elucidated. Here, we show the association of osmolytes and their consequent cooperative effects on the time-dependent turgor profile generated in a model cytosol consisting of KCl, D-Glc and L-Gln at experimentally measured plant motor/generic cell concentrations and at modified concentrations. We demonstrate the influence and association of the osmolytes using osmometry and NMR measurements. We also show, using a plant cell-inspired device we previously developed, that osmolyte complexes, rather than single osmolytes, permit to obtain higher turgor required by plant movements. We provide quantitative cues for deeper investigations of osmolyte transport for plant movement, and reveal the possibility of developing osmotic actuators exploiting a dynamically varying concentration of osmolytes.

Plant movements are enabled by the interplay between tissue structures and turgor pressure modulation^1^,^2^,^3^,^4^,^5^; the latter is related to osmotic pressure differences due to osmolyte concentration gradients in specific plant regions^1^,^6^,^7^. Plant cells are classically considered as fundamental “osmotic bricks”^8^ that are involved in turgor distributions and in which a special osmotic membrane^9^ surrounds the cytosolic osmolyte system. The osmotic membrane is provided by the cell wall and the plasma membrane. Typically, the cell wall is regarded to as a relatively diffusive matrix of polymers, whereas the plasma membrane constitutes the osmotic barrier. However, they act in tight physical contact^10^ and they carefully modulate, together, the mechanical strength of the cell wall and the selectivity of the plasma membrane^9^. The cytosolic osmolyte system is an aqueous gel solution with 200–300 mg·L^−1^ of proteins (e.g., glyceraldehyde-3-phosphate dehydrogenase, catalase, and ribosomal proteins) and 1–2 M small molecules (<900 kDa on average)^11^. Among the small molecules, previous genetic, biochemical and modelling studies^11^,^12^,^13^,^14^,^15^,^16^,^17^ have only considered potassium chloride (KCl) in relation to turgor dynamics: a 600 mM KCl concentration was measured in *Dionaea muscipula* and *Drosera glanduligera* active motor cells^1^, while ~250 mM KCl was found in non-motor cells^18^. Nevertheless, KCl has a low rejection coefficient (50–70%)^6^ and therefore cannot be efficiently retained in the cell^19^. This aspect makes it questionable whether KCl alone plays a major role in plant cell turgor generation/dynamics. In fact, other small molecules as D-Glc and L-Gln are detected at high concentration levels^18^,^20^,^21^ (50 mM for D-Glc^22^ and 30 mM for L-Gln^23^), having a higher rejection coefficient than KCl (99% for D-Glc^24^ and ~100% for L-Gln^25^). Nevertheless, their effect on turgor dynamics remains unclear.

To address this open issue, we considered a simplified plant cytosol containing relevant osmolytes. Specifically, starting from known plant osmolyte systems^16^,^21^, we considered KCl, D-Glc and L-Gln, as they are the most concentrated components in plant cytosol and have been shown to be closely related to turgor increases in motor cells^18^,^20^,^21^. Hence, we neglected the contributions of biomolecules (e.g., proteins, nucleic acids, and polysaccharides) due to their low concentrations (below 1 μM)^26^, and we neglected the contributions of drought stress osmolytes (e.g., ononitol and pinitol)^27^, as we were not addressing specific stress conditions. Moreover, we deliberately confined our attention to passive water transport across the plant cell boundary, since the complexity of a comprehensive study on aquaporins and water permeability of plant membranes^28^ was beyond the present scope.

Furthermore, in order to study osmosis-driven turgor dynamics, we leveraged a plant-inspired osmotic actuator^29^ we recently developed. This device features an actuation chamber containing the osmolyte solution, which is separated from a water reservoir by an osmotic membrane. The osmosis-driven water influx increases the actuation chamber volume by inducing the protrusion of an elastomeric bulging disk, which represents the compliant cell boundary and permits to transduce the actuation work. Bulging is directly related to the pressure in the actuation chamber, which corresponds to turgor. We designed the device so that actuation takes place on the minute timescale, by contextually matching the characteristic actuation time of an ideal giant plant cell having the same lengthscale as our actuator^29^ (i.e. 10 mm). In more detail, we matched the characteristic time by matching the volumetric stiffness typical of plant cells; to the purpose, we simultaneously acted on size and material properties of our device, by following a bioinspired approach (that is intrinsically different from, e.g., simple up-scaling)^29^. As a result, by recording the pressure in the actuation chamber over several characteristic times, we obtain turgor trends that are physically representative of the osmolytes behaviour *per se*, since the timescale intrinsically accounts for the experimental conditions (size and material properties of the cell-like device, and initial osmotic potential) in a physically consistent manner. We beg to remark that this is a rather unique advantage brought to the study by our device.

## Results

### Osmotic properties of the model cytosols

We prepared two physiologically relevant model cytosols based on the typical composition ratio of a non-motor cell (**M1**, KCl, D-Glc, L-Gln total concentration1 M) or of a motor cell (**M2**, KCl, D-Glc, L-Gln total concentration 1.5 M) (Table 1). We also introduced two modified cytosols with excess KCl (**M2a**) or excess D-Glc (**M2b**) to estimate the effect of an arbitrary increase in one component (the L-Gln concentration was kept fixed, as it is already near saturation, and the total concentration remains 1.5 M). Furthermore, we introduced additional derivative cytosols (**M1a-M1c**), each of which lacked one component. Finally, we introduced a 1.5 M KCl solution as a reference because of the key role attributed to this osmolyte in the literature (Table 1)^1^,^15^,^16^,^17^. The resulting pH of the model cytosols was in a neutral range, namely 7.0 ± 0.1, thus consistent with the 7.0–7.4 range reported in literature^30^ based on direct pH measurement in plant cell.

We studied the osmotic properties of the model cytosols by measuring the maximum osmotic pressure achievable using cryo-osmometry and by estimating its value as the sum of the single osmotic contributions of KCl, D-Glc and L-Gln (Table 1). For all the considered mixtures (except for **M1b**, which does not contain L-Gln), the measured osmotic potential was lower than the estimated value, suggesting that association phenomena are present among the osmolytes in solution. We computed the ratio between the measured and the estimated osmotic potential, hereafter labelled as osmotic potential ratio (Table 1), since the more it deviates from unity the larger the extent of the association should be. Based on this value, the considered osmolyte mixtures exhibited a non-ideal behaviour (mainly associated with L-Gln, as shown by the osmotic potential ratio for **M1b**). Let us observe that also the KCl osmotic potential ratio differed from unity, because of specific ion interactions occurring when the concentration is well above the 1 mM Debye–Hückel limit^31^. Moreover, we obtained a large deviation for **M1c**, for which association can only occur between two components, because specific ion interactions amongst K^+^, Cl^−^ and the zwitterionic form of glutamine can form ionic aggregates thus lowering the osmotic ratio^32^. We then focused on the three component model cytosols since they are more physiologically relevant. Assuming as a reasonable hypothesis that four-member supramolecular structures (containing D-Glc, L-Gln, K^+^ and Cl^−^) would form in aqueous solution at neutral pH, we elaborated the cryo-osmotic and calculated data to estimate the degree of osmolyte association, yielding estimates of approximately 20% for **M1, M2** and **M2a** and considerably lower (~7%) for **M2b** (Table 1).

### ^13^C NMR spectra of the model cytosols

**Figure 1:**
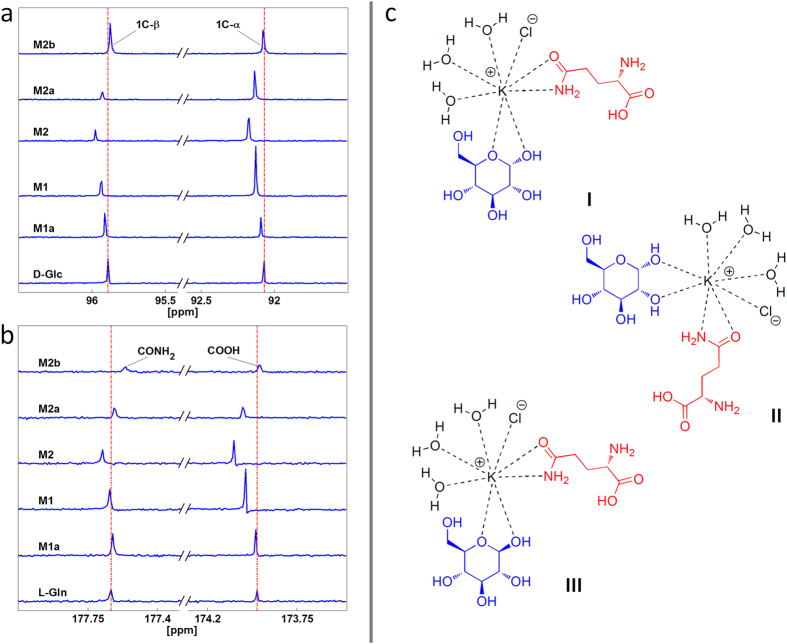
Detailed ^13^C NMR spectra of osmolyte mixtures and proposed supramolecular complexes. (**a)** Modification of the D-Glc chemical shift and intensity from that of the pure natural molecule in **M1a**, **M1**, **M2**, **M2a**, and **M2b**. Dashed vertical lines indicate the natural molecule peak as a reference. (**b)** Modification of the L-Gln chemical shift and intensity from that of the pure natural molecule in **M1a**, **M1**, **M2**, **M2a**, and **M2b**. Dashed vertical lines indicate the natural molecule peak as a reference. (Both for D-Glc and L-Gln, only the relevant spectral region is shown, for clarity, and the data are presented in a stacked plot with scaled intensity to show the modifications of the chemical shifts.) (**c)**. Proposed osmolyte complexes.

We studied the model cytosols by a comparative analysis of D-Glc and L-Gln ^13^C NMR spectra in the presence and absence of KCl in deuterated water. The modification of the D-Glc chemical shift and intensity from that of the pure natural molecule in **M1**, **M2**, **M2a**, and **M2b** is shown in Fig. 1a; corresponding results for the L-Gln are shown in Fig. 1b. We observed a modification of the chemical shift and intensity also for **M1a**; the corresponding results are shown in Fig. 1a,b in order to complement those of the three component cytosols that are of major interest for this study. All of the original ^13^C NMR spectra are reported in Supplementary Data S4.

As regards D-Glc in Fig. 1a, the anomeric C region in deuterated water is shown; the resonances at 92.1 and 95.9 ppm are from the C1α and C1β of glucose, respectively. The chemical shifts of these resonances change during D-Glc complexation with K^+^ due to modification of the magnetic environment of the D-Glc ^13^C nuclei. Based on these NMR signal intensity changes, interactions occurred between KCl ions and D-Glc that modified the mutarotation equilibrium (normally, the α:β ratio is 36:64) to favour α-D-Glc in the case of **M1** and **M2** (77:23 and 71:29, respectively) and β-D-Glc in the case of **M2a** (23:77) (Fig. 1a). Moreover, the interactions occurred in the anomeric C moiety, as we detected a chemical shift modification in all three component mixtures (**M1**, **M2**, **M2a**, **M2b**). As regards L-Gln in Fig. 1b, the carbonyl C region in deuterated water is shown; the resonances at 173.9 and 177.6 ppm are from the COOH and CONH_2_ of glutamine, respectively. The chemical shifts of these resonances change during L-Gln complexation with K^+^ due to modification of the magnetic environment of the L-Gln ^13^C nuclei. The observed modifications to chemical shifts and intensities in L-Gln ^13^C NMR spectra suggest that the interactions between KCl ions and L-Gln molecules correspond to the carbonyl moieties of COOH and CONH_2_ groups (Fig. 1b).

In light of the NMR results and by reviewing the crystal structures of similar complexes in the literature^33^,^34^, we postulated the osmolyte supramolecular structures shown in Fig. 1c, in which water molecules saturate all eight positions of the classic coordination sphere of K^+^ 33,34.

### Osmosis-driven expansion in the absence of external loads

**Figure 2:**
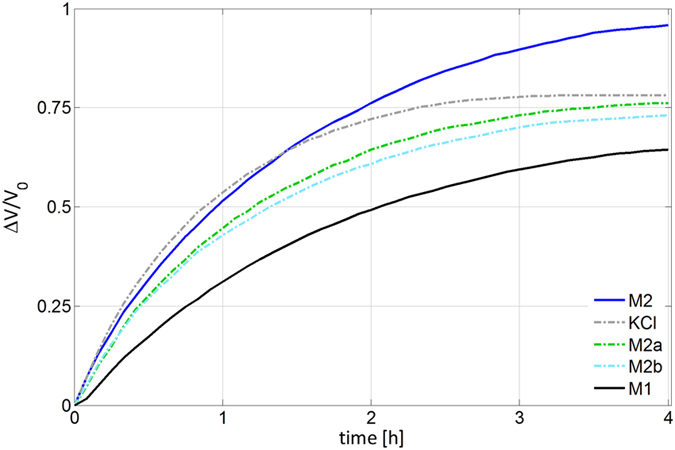
Osmosis-driven expansion in the absence of external loads. Relative variations in the volume of the chamber hosting the osmolyte mixture versus time (V_0_ represents the initial chamber volume) for KCl, **M1**, **M2**, **M2a** and **M2b**. For these experiments we used a modified version of our osmotic device^29^. Through these experiments (during which the pressure in the chamber was practically constant) we studied the behaviour of the model cytosols with respect to dynamic dilution. The model cytosol based on the typical composition ratio of plant motor cell (**M2**) outperformed the other osmolytes and in particular KCl: it more effectively sustained water influx over time in spite of the damping effect due to dilution.

We investigated the behaviour of the model cytosols under dynamic dilution by using a modified version of our osmotic device^29^. In more detail, we coupled the device to a frictionless piston to observe an osmosis-driven expansion not constrained by external loads. Let us remark that during these experiments we were not addressing turgor dynamics (the pressure in the actuation chamber, indeed, was practically constant); we aimed at a complementary characterization of the considered osmolytes. Fig. 2 shows the measured relative volume increase versus time. Let us also remark that the duration of these experiments was not dictated by physically relevant constraints, and thus we adopted a long observation window, so as to detect possible effects due to osmolyte dilution as long as possible. Noticeably, the model cytosol based on the typical composition ratio of plant motor cell (**M2**) outperformed the other osmolytes, including KCl. Moreover, its trend was very similar to the one of the other plant-inspired cytosol (**M1)**, which cannot directly compete in this experiment with the other osmolytes because of its lower total molarity.

### Osmosis-driven turgor dynamics

**Figure 3:**
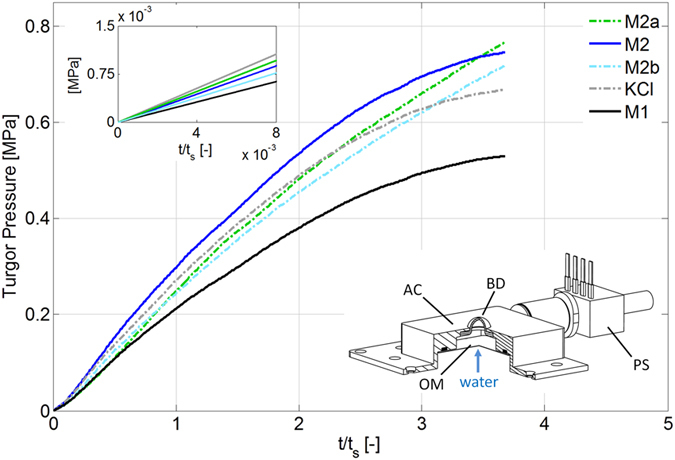
Osmosis-driven turgor dynamics. Main panel: turgor versus time, as measured by instrumenting the osmotic actuator^29^ with a pressure sensor, for KCl, **M1**, **M2**, **M2a** and **M2b**. Through these experiments (during which the pressure in the actuation chamber increased because of the osmosis-driven water influx) we directly measured turgor dynamics. Time axis is non-dimensionalised by using the characteristic time t_s_ of the actuator, so as to intrinsically account for the experimental conditions and obtain turgor trends that are physically representative of the osmolytes behaviour *per se*. The three component mixtures at 1.5 M (**M2**, **M2a** and **M2b**) outperformed KCl at the same molarity. During most of the observation period; the model cytosol based on the typical composition ratio of plant motor cell (**M2**) was the most effective at turgor formation. **Top inset:** initial recordings showing that the initial osmotic potential determines the turgor formation rate at the very beginning of the experiments (yet alone it is not sufficient to rank the mixtures with respect to turgor formation over time). **Bottom inset:** schematic of the osmotic actuator as used in these experiments (AC: actuation chamber; OM: osmotic membrane; BD: bulging disk; PS: pressure sensor).

We measured the osmosis-driven turgor dynamics by using the osmotic actuator^29^ (bottom inset in Fig. 3). As anticipated, our osmotic actuator features a characteristic actuation time t_s_ matching that of an ideal giant plant cell, and by accounting for this timescale we obtained turgor trends physically representative of the osmolytes behaviour *per se*. The results are shown in Fig. 3, in which time is consistently non-dimensionalised by means of t_s_. These results show that the three component mixtures at 1.5 M (**M2**, **M2a** and **M2b**) outperformed KCl at the same molarity. During most of the observation period, the model cytosol based on the typical composition ratio of plant motor cell (**M2**) was the most effective at turgor formation (and also in this experiment it featured a trend very similar to that of **M1**). Moreover, these results show that the initial osmotic potential determined the turgor formation rate at the very beginning of the experiments (top inset in Fig. 3), yet alone it is not sufficient to rank the mixtures with respect to effective turgor formation over time.

## Discussion

Based on the known rejection coefficients of KCl, D-Glc and L-Gln, which are the most concentrated components in plant cytosol, we questioned whether KCl alone could play a major role in plant cell turgor modulation as suggested by previous studies. We therefore introduced some simplified cytosols based on KCl, D-Glc and L-Gln: they permitted to contain the complexity of the present study while keeping physical representativeness. The pH of the model cytosols was fully consistent with plant cell measurements: this supported the subsequent characterization, in particular the NMR study.

The osmometry results suggested the presence of association phenomena among the osmolytes in solution and highlighted the non-ideal character of the osmolyte mixtures, mainly due to L-Gln (as observed through the two component derived mixtures). The derived association degree and the osmotic potential ratio provided a preliminary estimate of osmolytes cooperation, which was confirmed by NMR. In particular, the ^13^C NMR data demonstrated that anomeric C atoms in D-Glc and carbonyl C atoms in L-Gln are close to K^+^ in the supramolecular complexes formed in solution, for which we postulated three structures (Fig. 1c) based on crystal structures in the literature. According to the postulated structures, potassium ions are complexed by D-Glc and L-Gln in a geometry that is comparable to that of other alkaline metals, sugars and amino acid crystals. Moreover, the size of the resulting complex is larger than for the single ions (K^+^ and Cl^−^). Furthermore, water completes the metal coordination number, and the structures I, II, and III in Fig. 1c, which are equally probable, suitably describe the potassium ion coordination in the considered three component model cytosols. Let us finally observe that the presence of L-Gln supports the stability of the considered complexes, and therefore we rightfully considered the three component model cytosols for the subsequent experiments.

The osmosis-driven expansion in the absence of external loads did not address turgor dynamics; rather it aimed at a complementary characterization of the osmolyte mixtures as their behaviour under dynamic dilution. The fact that dilution degraded the initial osmotic potential less effectively for **M2** can be explained in terms of a cooperative effect due to osmolyte association. Specifically, the disassembly of supramolecular structures due to dilution increases the osmolyte availability in solution, thus counteracting the decrease in concentration caused by water influx. This effect, which occurs for all mixtures, was particularly apparent for the model cytosol based on the typical composition ratio of plant motor cell (**M2**), which outperformed KCl alone (Fig. 2).

The results in Fig. 3 directly address turgor dynamics, and it is worth remarking that we measured turgor pressures that reached nearly 1 MPa, thus spanning the relevant range for plant movement^35^. At the very beginning, the turgor increased fastest for KCl; recalling the classical Van ’t Hoff law, this result is consistent with the fact that KCl had the greatest initial osmotic potential (Table 1). However, the initial osmotic potential just provides an initial “snapshot” of the story: it is not sufficient, alone, to rank the turgor formation performance of the mixtures. The fact that the model cytosols at 1.5 M, and in particular that based on the plant motor cell (**M2**), outperformed KCl in turgor output can still be explained in terms of the cooperative effect of osmolyte association, which can decrease osmolyte backflow through the (pressurized) osmotic membrane thanks to the larger size of complexes. Hence, supramolecular structures obtained by osmolyte association can sustain water influx and the consequent generation of turgor over time. Indeed, K^+^ and Cl^−^ have Stokes radii (0.12–0.14 nm)^36^ of the same order as the membrane pore size (0.3–0.5 nm)^37^ and are therefore already prone to backflow by diffusion. Let us remark that the difference in osmolytes “ranking” between Figs 2 and 3 is consistent with the different nature of the underlying experiments: the previously mentioned size effect, in particular, plays a stronger role in the experiment tackling turgor dynamics, where the actuation chamber is properly pressurized thus potentially promoting backflow^38^.

We clearly leveraged the osmotic actuator: the turgor trends in Fig. 3 are physically representative of the osmolytes behaviour *per se*, and this achievement was enabled by our cell-like device. However, we are aware of the simplifications brought by this device. In particular, we used a single artificial membrane made of cellulose for representing both the plant cell wall and the plasma membrane. We chose a single membrane, robust enough to resist high turgor pressure and permeable not to hamper solute transport, in order not to introduce technological challenges incommensurate with the current developmental stage. Nevertheless, the adopted osmotic membrane is representative of its biological counterpart in terms of permeability and strength^9^,^10^,^39^. Let us also observe that we did not consider artificial phospholipid membranes or specific dialysis membranes in place of the adopted cellulose membrane. Indeed, available phospholipid membranes are currently too small (order of 100 μm) and not robust enough to bear the sought turgor pressures^40^,^41^. Moreover, the molecular cut off of the available dialysis membranes is too high for them to properly reproduce the plant osmotic membrane, in particular as regards KCl rejection.

Furthermore, we could not address active osmolyte transport. However, the plasma membrane features a pore size (0.3–0.4 nm^28^) comparable to that of the adopted osmotic membrane, and it deforms during plant actuation^39^ thus favouring potential osmolyte backflows. Hence, supra molecular complex formation could affect how the solutes get actively transported across the plasma membrane as well, or diffuse through the channel pores. As a result, the previously mentioned size exclusion limit can be relevant for active osmolyte transport, and the osmolyte cooperation that we originally observed in this study can effectively affect turgor dynamics in real plant cells.

## Conclusion

This study sheds light on the role played by osmolytes as a pool (rather than KCl alone) in turgor dynamics, as observed for both physiologically relevant and modified osmolyte mixtures. Our results provide quantitative cues for plant scientists who investigate turgor mechanisms, still debating fundamental aspects^42^. Moreover, they reveal the possibility of using plant-inspired osmolytes such as **M1** and **M2** in novel actuation devices that harness biomolecular systems to actively regulate actuation performance. Our study provides a remarkable example of closing the loop between science and technology. Indeed, the development of our osmosis-based device was inspired by the study of plant cell actuation, and here it provides a unique experimental platform for advancing the quantitative investigation of plant actuation mechanisms.

## Methods

### Model cytosols preparation

Potassium chloride, α-(d)-glucose and l-glutamine were purchased from Sigma-Aldrich; osmolyte solutions were prepared using deionised water and were stored overnight at 4 °C before use. The solution pH was measured by means of a pH meter (Eutech Instruments, WP 600 series meter, model PC 650) in order to detect potential variations with respect to the neutral value due to the presence of glutamine (because of its ionisation in water, as amino acid).

### Osmometric and NMR measurements

Osmotic pressure measurements (n = 5) were performed using an OSMOMAT 030 cryo-osmometer (Gonotec, Berlin, Germany). ^13^C NMR measurements were performed using a Bruker 400-MHz NMR spectrometer (Bruker, Bremen, Germany) with deuterated water. All of the original ^13^C NMR spectra are reported in Supplementary Data S4.

### Estimate of the osmotic potential

The osmotic potential of the considered mixtures was estimated as the sum of the contributions due to each component. Each contribution was estimated by using the following expression (namely a modification of the classical Van’t Hoff law^1^):


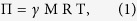


where M denotes the osmolyte molarity, R = 8.314 J K^−1^ mol^−1^ is the universal gas constant, T denotes the absolute temperature, and γ is a factor globally accounting for deviations from the ideal behaviour. In particular, α-(d)-glucose can be classically considered as ideal, so that we adopted γ = 1. Moreover, for KCl we adopted γ = φ i, where i = 2 is the classical Van’t Hoff factor (depending on the degree of osmolyte dissociation) and φ = 0.90 is an empirical correction^43^. Finally, l-glutamine exhibits a non-ideal behaviour that can be described by adopting^44^:


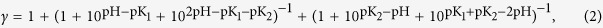


where pK_1_ = 2.17 and pK_2_ = 9.13 denote the ionisation constants for l-glutamine^45^, and pH = 7.0 is the measured solution pH.

### Measurement of the osmosis-driven turgor dynamics

A schematic of the osmotic actuator is reported in Fig. 3 (bottom inset); full details on its design and implementation can be found in previously published work^29^,^46^. In particular, the osmotic membrane is a semi-permeable forward-osmosis membrane (HTI, Hydration Technology Innovations, Scottsdale, AZ, USA), made of cellulose triacetate that is cast onto a non-woven backing consisting of polyester fibres that are individually coated with polyethylene. Its water permeability coefficient is 3·10^−13^ m s^−1^ Pa^−1^, and it is optimized to work with glucose and salt solutions. Besides properly acting as osmotic barrier, the chosen osmotic membrane sustains high pressure differentials without appreciable deformations, as needed for our purposes, thanks to devoted mechanical supports: we accurately verified that the osmotic actuator behaviour is fully reproducible for turgor pressures of up to 1 MPa. Also based on the adopted elastomeric bulging disk (PARA natural rubber, SIGAP, Italy), the osmotic actuator features a characteristic actuation time on the order of a minute. For completeness, the characteristic actuation time scales as follows^29^:


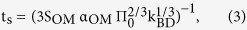


where S_OM_ and α_OM_ respectively denote the surface area and the permeability of the osmotic membrane (the former indirectly brings the device lengthscale into play), П_0_ is the initial osmotic potential, and k_BD_ is the stiffness of the bulging disk (accounting for both elastic and geometrical properties of the disk). For each osmolyte, we estimated t_s_ by means of Eq. (3), using in particular the measured value of П_0_ (Table 1). The actuator was equipped with a differential pressure sensor (24PCGFA6D, Honeywell Sensing and Control, France) with a 1.72 MPa maximum pressure and 10^−2^ MPa sensitivity. After loading the actuation chamber with the chosen osmolyte, we recorded turgor pressure over nearly 4 characteristic times. All tests were performed in triplicate; error bars (standard deviation) are reported in Supplementary Fig. S2. The experimental platform is shown in Supplementary Fig. S3.

### Measurement of the osmosis-driven expansion in the absence of external loads

We slightly modified the osmotic actuator in order to use it for measuring the osmosis-driven expansion of the actuation chamber in the absence of external loads. In particular, we replaced the bulging elastomeric disk with a piston sliding within a frictionless guide. In the adopted configuration, the osmosis-driven water influx increases the actuation chamber volume by inducing the piston displacement, which occurs at constant pressure (namely the external, atmospheric pressure). After loading the actuation chamber with the chosen osmolyte, we recorded piston displacement over four hours in order to assess the osmolyte behaviour with respect to dynamic dilution. Let us remark that, since the osmotic device is not used in its standard configuration, it does not set any characteristic observation times in this experiment; hence, we simply observed the osmosis-driven actuation over a long period, so as to detect possible effects due to osmolyte dilution as long as possible. Piston displacement was recorded with a camera (D40, Nikon, Japan) and processed by standard image processing (Matlab, Mathworks, Natick, MA, USA). All tests were performed in triplicate; error bars (standard deviation) are reported in Supplementary Fig. S1.

## Additional Information

**How to cite this article**: Argiolas, A. *et al*. Osmolyte cooperation affects turgor dynamics in plants. *Sci. Rep.* **6**, 30139; doi: 10.1038/srep30139 (2016).

## Figures and Tables

**Table 1:** Measured osmotic potentials and derived osmolyte association degrees.

Osmolyte mixture	Composition [KCl]:[D-Glc]:[L-Gln]	Total molarity (M)	Measured osmotic potential П_0_ (MPa)^a^	Estimated osmotic potential (MPa)	Osmotic potential ratio^b^	Association degree (%)^c^
M1	5:12:3	1.00	2.93 ± 0.02	3.35	0.87	19.5
M1a	0:12:3	0.75	1.95 ± 0.04	2.23	0.87	—d
M1b	5:12:0	0.85	2.65 ± 0.02	2.62	1.01	—d
M1c	5:0:3	0.40	1.44 ± 0.01	1.87	0.77	—d
M2	15:12:3	1.50	4.88 ± 0.02	5.56	0.88	18.0
M2a	25:12:3	1.50	4.98 ± 0.02	5.84	0.85	22.5
M2b	15:22:3	1.50	4.83 ± 0.01	5.11	0.95	7.5
KCl	—	1.50	6.37 ± 0.01	6.74	0.95	—d

^a^Mean ± std over 5 repetitions.

^b^Ratio between measured and estimated osmotic potential.

^c^Calculated considering a complex between KCl, glutamine and glucose (n = 4).

^d^Association is proposed for the three component mixtures.
